# Anti-IL-6 Receptor Antibody Causes Less Promotion of Tuberculosis Infection than Anti-TNF-*α* Antibody in Mice

**DOI:** 10.1155/2011/404929

**Published:** 2011-02-22

**Authors:** Masaji Okada, Yoko Kita, Noriko Kanamaru, Satomi Hashimoto, Yasushi Uchiyama, Masahiko Mihara, Yoshikazu Inoue, Yoshiyuki Ohsugi, Tadamitsu Kishimoto, Mitsunori Sakatani

**Affiliations:** ^1^Clinical Research Center, National Hospital Organization Kinki-Chuo Chest Medical Center, Osaka 591-8555, Japan; ^2^Chugai Pharmaceutical Co., Ltd., Product Research Department, Shizuoka 412-8513, Japan; ^3^Laboratory of Immune Regulation, Graduate School of Frontier Biosciences, Osaka University, Osaka 565-0871, Japan

## Abstract

*Objective*. Our aim was to investigate the effects of IL-6 blockade on the progression of *Mycobacterium tuberculosis* (TB) and compare them with those of TNF-*α* blockade in mice. *Methods*. Mice were intravenously infected with TB and injected with antibodies. Survival was monitored and histological and immunological studies were carried out. *Results*. All anti-IL-6R Ab-treated mice and 8 of 10 control mice survived until sacrificed 224 days after TB challenge, whereas anti-TNF-*α* Ab-treated mice all died between 120 and 181 days. Anti-IL-6R Ab-treated mice exhibited no significant differences in TB CFU in organs, including the lungs, and no deterioration in histopathology compared to control mice at 4 weeks. In contrast, anti-TNF-*α* Ab-treated mice exhibited increased TB CFU and greater progression of histopathological findings in organs than control mice. Spleen cells from anti-TNF-*α* Ab-treated mice had decreased antigen-specific response in IFN-*γ* release and proliferation assays. The results in anti-IL-6R Ab-treated mice suggest that spleen cell responses were decreased to a lesser degree. Similar results were obtained in IL-6 knockout (KO) mice, compared with TNF receptor 1 (TNFR1) KO and TNFR1/IL-6 double KO (DKO) mice. *Conclusion*. IL-6R blockade promotes the progression of TB infection in mice far less than TNF-*α* blockade.

## 1. Introduction

Unregulated cell-mediated immunity may lead to chronic autoimmune inflammatory diseases such as rheumatoid arthritis (RA). Proinflammatory cytokines such as TNF-*α* and IL-6 play important roles in the development and progression of these diseases. As a result, TNF-*α* blockers, such as infliximab and etanercept, and the anti-IL-6 receptor (IL-6R) antibody (Ab) tocilizumab(TCZ) have exhibited efficacy superior to conventional DMARDs in the treatment of RA [1–6]. One concern with anti-TNF-*α* Ab therapy, however, is that TNF-*α* is essential for protection against *Mycobacterium tuberculosis* (TB), and it has, in fact, been reported that anti-TNF-*α* Ab therapy is associated with reactivation of tuberculosis [7–11]. As is well known, TB grows inside macrophages and is killed by activated macrophages. Granuloma formation is critical in preventing TB infection in which TNF plays central roles [11]. It is, therefore, thought that inhibition of granuloma formation is the major mechanism of reactivation of TB in patients treated with TNF blockers. 

In contrast, no published study has indicated that IL-6 plays roles in granuloma formation. Accordingly, it is expected that patients treated with TCZ will not undergo reactivation of TB. In fact, one study found that the incidence of TB reactivation in TCZ-treated patients did not differ from that in controls [12]. However, T cell-mediated immunity also plays a major role in protecting infected hosts from TB. Th1 cells are induced by IL-12 to secrete IFN-*γ*, which works with IL-2 and IL-6 to induce cytotoxic T cells, which produce granulysin, leading to the death of TB inside of macrophages. Thus, IL-6 is of critical importance for acquired immunity to TB [13]. In fact, it has been reported that IL-6 blockade increased susceptibility to tuberculosis in IL-6-deficient mice [14] and anti-IL-6 antibody-treated mice [15]. However, no reports describing the effects of anti-IL-6R Ab on the immune mechanisms involved in protection against TB infection have been published. 

Clinically, it is particularly important to determine to what extent IL-6 blockade by anti-IL-6R antibody promotes TB infection. 

 This paper describes experiments conducted to investigate and compare the effects of IL-6 and TNF-*α* blockade on the development of TB infection in mice by examining various indicators of disease in TB-challenged mice treated with antibodies to IL-6R and TNF-*α* and also in TB-challenged IL-6 knockout (KO), TNF-*α* receptor 1 (TNFR1) KO, and TNFR1/IL-6 double-KO (DKO) mice.

## 2. Materials and Methods

### 2.1. Animals

Female BALB/c and DBA/1 mice were purchased from Clea Japan (Tokyo, Japan) and Japan SLC (Shizuoka, Japan), respectively. IL-6 KO, TNFR1 KO, and TNFR1/IL-6 DKO mice (backcrossed with DBA/1 mice) were kindly provided by Dr. Y. Saeki, Osaka University (Osaka, Japan) [16]. 

 The mice were raised under specific pathogen-free conditions, maintained in isolator cages, manipulated in laminar flow hoods, and used between 8 and 10 weeks of age. After infection with TB, the animals were housed in individual microisolator cages in a Bio-safety Level (BSL) 3 animal facility.

### 2.2. Reagents and Antibodies

Purified protein derivative of tuberculin (PPD) was obtained from Japan BCG (Tokyo, Japan). Killed TB H37Ra (referred to as “killed TB” below) was obtained from Difco Laboratories (Detroit, MI, USA), and foetal calf serum was obtained from HyClone (Logan, UT, USA). 

 Rat anti-murine IL-6R Ab (clone: MR16-1) was prepared by Chugai Pharmaceutical (Tokyo, Japan) [17]. Hamster monoclonal anti-mouse TNF-*α* Ab (clone: TN3-19.12), which has been shown to neutralize murine TNF-*α* in vivo [18], was obtained from Techne (Minneapolis, MN, USA). 

 Purified rat IgG was obtained from ICN Pharmaceuticals (Aurora, OH, USA) and used as the control Ab for anti-IL-6R Ab (referred to below as “control Ab 1”). Hamster IgG was purchased from Rockland (Gilbertsville, PA, USA) and used as the control Ab for anti-TNF-*α* Ab (“control Ab 2”).

### 2.3. Bacteria

TB H37Rv was kindly provided by Dr. I. Sugawara (JATA, Tokyo, Japan). A single colony was grown by a method previously reported [19, 20].

### 2.4. Challenge Infection of Animals and Bacterial Load Determination

The mice were challenged i.v. with 5 × 10^5^ colony forming units (CFU) of TB, and their survival was monitored daily for 224 days. At 4 and 32 weeks after challenge, the lungs, spleen, and liver were removed aseptically and homogenized. Serial dilutions were plated on agar, and the TB CFU count 14 days later was determined by a method previously reported [19, 20].

### 2.5. Administration of Antibodies

**Figure 1:**
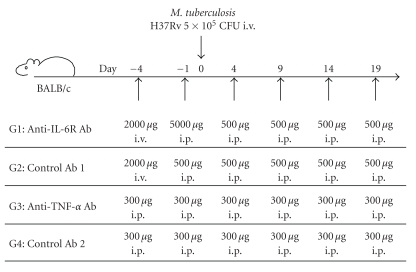
Protocol of experiment. Mice were treated i.p. or i.v. with antibodies and injected with live TB. Each group consisted of 5 mice.

BALB/c mice were injected with anti-TNF-*α* Ab or control Ab 2 (300 *μ*g/mouse i.p.) 4 days and 1 day before challenge and 4, 9, 14, and 19 days after challenge, or with anti-IL-6R Ab or control Ab 1 4 days before challenge (2000 *μ*g i.v.) and then 1 day before challenge and 4, 9, 14, and 19 days after challenge (500 *μ*g i.p.) (Figure 1). The regimen of administration for anti-TNF-*α* Ab and anti-IL-6R Ab was modified as described in previously published papers [21, 22].

### 2.6. Histopathological Analysis

Lung and liver tissues from the mice were fixed with 10% buffered formalin and embedded in paraffin. Each block was cut into 4-*μ*m-thick sections and stained using haematoxylin and eosin. Semiquantitative morphometric analysis of the pathologic slides was performed using a microscope equipped with a micrometer by our modification of the method of Dascher et al. [23]. In brief, the long and short axes of each pathologic TB lesion including granuloma in the field (×40 magnification) were measured, multiplied, and summed. Three random fields from each tissue section were evaluated, and the average score of the fields was designated the pathologic TB lesion index (×10^−2^ mm^2^).

### 2.7. Proliferative Response of Spleen Cells

To examine T cell proliferation in response to TB antigen (PPD), spleen cells (1 × 10^5^) obtained 4 weeks after challenge were cultured in a 96-well flat bottom plates (Linbro; Flow Laboratories, McLean, VA, USA) in the presence of PPD (20 *μ*g/mL) for 60 h at 37°C, and then pulsed with 20 *μ*L of BrdU per well for the final 2 h of incubation. After removal of the culture medium, the cells were fixed and the DNA denatured by adding FixDenat, and cell proliferation was quantified based on measurement of BrdU incorporated during DNA synthesis using a Cell Proliferation ELISA kit (Roche Diagnostic Corp., Indianapolis, IN, USA) as an indicator of proliferation.

### 2.8. Cytokine Production in TB-Specific Spleen Cells

To examine TB-specific T cell immune reactivity, spleen cells obtained 4 weeks after challenge were cultured in triplicate in 2 mL of medium (2.5 × 10^6^ cells/mL) in the presence of PPD (20 *μ*g/mL). Culture supernatant was collected 48 h later, and the levels of IFN-*γ* and IL-6 were measured using sandwich ELISA kits (BD Opt EIA; BD Biosciences Pharmingen) according to the manufacturer's instructions [19]. 

## 3. Results

### 3.1. Survival of Ab-Treated Mice after TB Challenge

**Figure 2:**
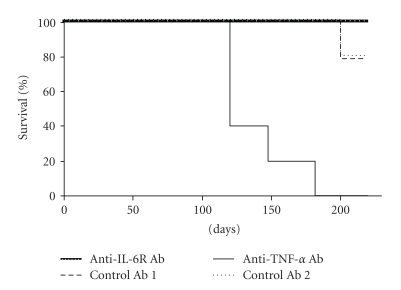
Survival of TB-challenged BALB/c mice treated with antibodies to TNF-*α* and IL-6R. Mice were injected with TB and antibodies, as shown in Figure 1, and monitored daily for survival. Each group consisted of 5 mice.

Of the 5 mice in each control group, 1 mouse in each group died by 202 days after TB infection (Figure 2). None of the 5 mice in the anti-IL-6R Ab group died by day 224, indicating that anti-IL-6R Ab treatment did not affect survival. In contrast, all of the 5 anti-TNF-*α* Ab-treated mice died between 120 and 181 days after TB challenge, resulting in significantly shorter survival in the anti-TNF-*α* Ab-treated group (*P* < .01).

### 3.2. Confirmation of IL-6 Blockade

Since it has been reported that anti-IL-6R Ab-treatment significantly reduced serum amyloid A (SAA) production [24], we measured SAA 4 weeks after TB infection to confirm blockade of IL-6 signalling by anti-IL-6R Ab. Production of SAA was almost completely suppressed in anti-IL-6R Ab-treated mice. In contrast, it was augmented significantly in anti-TNF-*α* Ab-treated mice (Table 1).

### 3.3. TB CFU in Organs from Ab-Treated Mice

**Figure 3:**
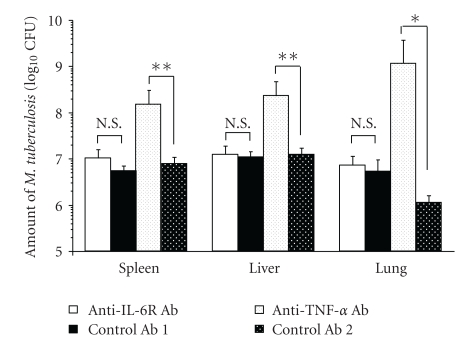
Growth of TB in lungs, spleen, and liver of Ab-treated mice 4 weeks after infection. TB CFU counts in lungs, spleen, and liver were determined 4 weeks after infection. Results are expressed as the mean ± S.E.M. of the 5 mice in each group. *P < .05, **P < .01 by Student's *t*-test. N.S.: Not significant by Student's *t*-test.

There were no significant differences in TB CFU count in the lungs, spleen, or liver between anti-IL-6R Ab-treated and control Ab 1-treated mice 4 weeks after TB challenge. In contrast, anti-TNF-*α* Ab-treated mice had significantly higher TB CFU than control Ab 2-treated mice in the lungs (*P* < .05), liver (*P* < .01), and spleen (*P* < .01) 4 weeks after TB challenge (Figure 3). CFU count in the lung was higher in control Ab 1 than control Ab 2, though not to a significant extent (Figure 3).

### 3.4. Histopathological Findings in Ab-Treated Mice

**Figure 4:**
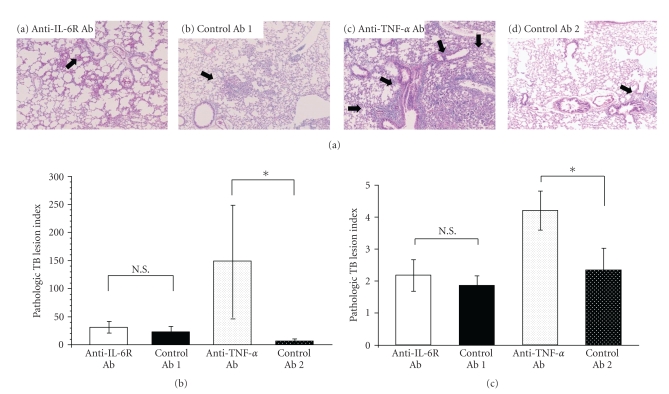
Histopathological analysis of mice 4 weeks after TB challenge. (a) Representative photomicrographs of lung tissues sections harvested from mice administered (a) anti-IL-6R Ab, (b) control Ab 1 (IL-6R), (c) anti-TNF-*α* Ab, or (d) control Ab 2 (TNF-*α*). Arrows indicate TB lesions including poorly defined granuloma-like lesions (×40, hematoxylin-eosin stain). (b) Pathologic TB lesion index in lungs. Results are the mean ± S.E.M. of triplicate samples from 5 mice per group. *P < .05 by Student's *t*-test. N.S.: Not significant by Student's *t*-test. (c) Pathologic TB lesion index in liver. Results are the mean ± S.E.M. of triplicate samples from 5 mice per group. *P < .05 by Student's *t*-test. N.S.: Not significant by Student's *t*-test.

When lungs and livers obtained from Ab-treated mice 4 weeks after TB challenge were examined, the lungs from mice treated with anti-IL-6R Ab (Figure 4(a)-a) and the corresponding control (Figure 4(a)-b) had a few TB lesions including poorly defined granuloma-like lesions and mononuclear cell infiltration. In contrast, lungs from mice treated with anti-TNF-*α* Ab (Figure 4(a)-c) had more and larger TB lesions including poorly-defined granuloma-like lesions, with more significant inflammation involving mononuclear cells, neutrophils, and histiocytes than did mice of the corresponding control (Figure 4(a)-d). 

 Anti-TNF-*α* Ab-treated mice, but not anti-IL-6R Ab-treated mice, exhibited a significant increase in the pathologic TB lesion index in lungs (Figure 4(b)) and liver (Figure 4(c)) compared with the corresponding controls (*P* < .05).

### 3.5. Cytokine Production by Spleen Cells from BALB/c Mice 4 Weeks after TB Challenge

To examine TB-specific T cell immune reactivity, spleen cells were cultured in the presence of PPD for 48 h, and T cell activity was assessed by the amount of IFN-*γ* in culture supernatant. It is known that helper T cells are mainly responsible for the findings obtained in this assay system [25]. 

**Figure 5:**
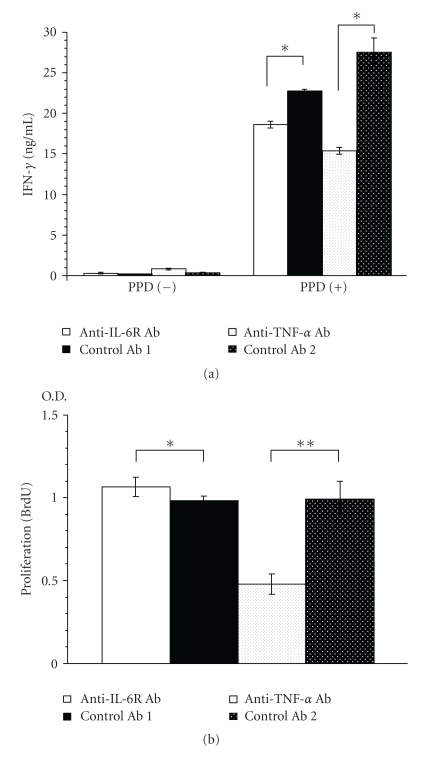
IFN-*γ* production and proliferation of spleen cells obtained 4 weeks after TB infection from Ab-treated mice in response to TB antigens (PPD). (a) Spleen cells from each group (*n* = 5) were pooled and cultured with 20 *μ*g of PPD or TB antigens per mL for 48 h, and then IFN-*γ* in the culture supernatant was measured. Results are the mean ± S.E.M of triplicate culture. *P < .01 versus corresponding control by Student's *t*-test. (b) Spleen cells were incubated in the presence of PPD, and BrdU incorporation in the nucleus was measured as O.D. ***(optical density at 340 nm) as an indicator of proliferation. Results are the mean ± S.E.M. of triplicate samples from 5 mice per group. *P < .05 and **P < .01 versus corresponding control by Student's *t*-test.

 In the presence of PPD, splenic T cells from anti-IL-6R Ab-treated mice produced less IFN-*γ* (18% less) and splenic T cells from anti-TNF-*α* Ab-treated mice produced much less IFN-*γ* (44% less) than the corresponding controls (Figure 5(a)).

### 3.6. Proliferation of Spleen Cells from TB-Infected BALB/c Mice

When spleen cells were cultured in the presence of PPD to examine T cell proliferation in response to TB antigen, anti-IL-6R Ab did not greatly affect the proliferation of spleen cells (8% increase), whereas anti-TNF-*α* Ab treatment strongly inhibited spleen cell proliferation (52% decrease) compared with the corresponding control (Figure 5(b)).

### 3.7. Survival of IL-6 KO and TNFR1 KO DBA/1 Mice after TB Challenge

**Figure 6:**
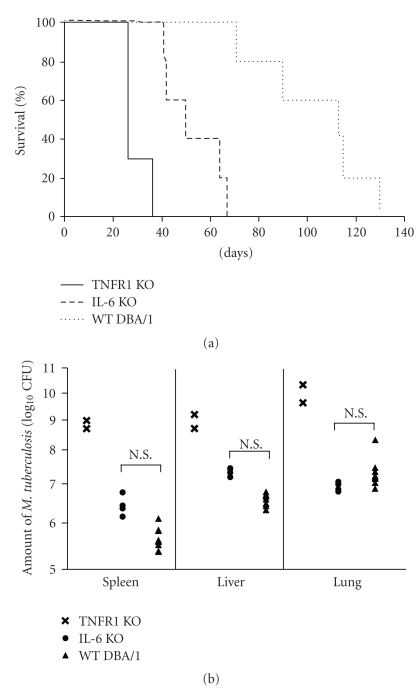
Survival and TB CFU in spleen, lungs, and liver of TB-challenged IL-6 KO, TNFR1 KO, and WT DBA/1 mice. (a) Survival of TB-infected IL-6 KO mice (*n* = 5), TNFR1 KO mice (*n* = 3), and WT DBA/1 control mice (*n* = 10). IL-6 KO mice versus TNFR1 KO mice (*P* < .01, Kaplan-Meyer analysis). (b) TB CFU in lungs, spleen, and liver 4 weeks after infection in IL-6 KO mice (*n* = 4), TNFR1 KO mice (*n* = 2), and WT DBA/1 control mice (*n* = 10). Results are expressed as the mean ± S.E.M. For TNFR1 KO mice, individual values are shown. N.S.: Not significant by Student's *t*-test.

The median survival times of wild-type (WT) control mice, IL-6 KO mice, and TNFR1 KO mice were 112, 47, and 26 days, respectively (Figure 6(a)). Kaplan-Meyer analysis demonstrated that both types of KO mice died earlier than the WT control mice (*P* < .01), and that TNFR1 KO mice died even earlier than IL-6 KO mice (*P* < .01).

### 3.8. TB CFU in Organs from IL-6 KO and TNFR1 KO DBA/1 Mice after TB Challenge

There were no significant differences between IL-6 KO and WT control mice with respect to TB CFU counts in the lungs, spleen, or liver 4 weeks after TB challenge. In contrast, 3 of the 5 TNFR1 KO mice died by the time of assay and both of the remaining 2 mice exhibited increased TB CFU in all 3 types of organs (Figure 6(b)).

### 3.9. Cytokine Production in Spleen Cells from KO Mice 4 Weeks after TB Challenge

**Figure 7:**
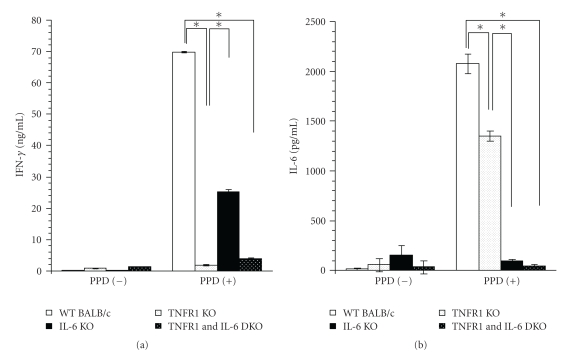
Cytokine production of PPD-stimulated spleen cells from IL-6 KO, TNFR1 KO, TNFR1 and IL-6 DKO, and WT BALB/C mice. Single cell suspensions obtained from each group 4 weeks after infection in IL-6 KO mice (*n* = 4), TNFR1 KO mice (*n* = 2), and WT DBA/1 control mice (*n* = 10) were pooled and cultured with 20 *μ*g/mL of PPD for 48 h, and then cytokine concentrations were measured. Results are means ± S.E.M. of triplicate cultures in each group. *P < .01 by Student's *t*-test.

When stimulated with PPD, spleen cells from IL-6 KO mice produced less IFN-*γ* than the WT control, but TNFR1 KO and DKO mice produced much less IFN-*γ* than IL-6 KO mice. Similarly, spleen cells from TNFR1 KO mice produced somewhat less IL-6 than the WT control, whereas IL-6 production was almost completely suppressed in IL-6 KO and DKO mice (Figure 7).

## 4. Discussion

The experiments described here examined whether IL-6R blockade influences the course of TB infection in BALB/c mice and compared the effects of IL-6 blockade with those of TNF-*α* blockade in the same experiments performed at the same time. Our findings clearly demonstrate that anti-IL-6R Ab treatment increases the susceptibility of these mice to TB infection to some extent, but that this increase is far less marked than that induced by anti-TNF-*α* Ab treatment. This suggests that the involvement of IL-6 in protection from TB infection is much less important than that of TNF-*α*. To the best of our knowledge, this is the first time such findings have been reported, and they have extremely important clinical implications since they suggest that anti-IL-6R Ab treatment will not often cause the reactivation of TB in RA patients, an issue that has limited the clinical use of TNF-*α* blockers. In fact, a recently published study found that the incidence of TB infection in patients treated with TCZ was low [12]. In contrast, many studies have found that patients treated with TNF blockers suffered reactivation of TB [7–11]. These clinical findings reflect the results obtained here in mice.

After TB challenge, there were no differences between anti-IL-6R Ab-treated and control Ab-treated mice with respect to survival (Figure 2) or the proliferation of TB in the lungs, liver, or spleen 4 weeks after infection (Figure 3). Furthermore, both groups had a few TB lesions including poorly defined granuloma-like lesions (arrows) in the lungs (Figure 4), and there was no significant difference between them in the proliferation of splenic T cells in response to TB antigen (Figure 5(b)).

On the other hand, comparison of IFN-*γ* production levels showed that there was less PPD-stimulated TB-specific splenic helper T cell activity in the anti-IL-6R Ab-treated mice than in control Ab-treated mice (Figure 5(a)). The finding that survival was not affected by anti-IL-6R Ab treatment shows that the decreases in T cell function and IFN-*γ* production were not large enough to affect the survival of the TB-infected mice. It is not surprising that IL-6 blockade only weakly inhibited immune reactivity since, in addition to IL-6 [26, 27], various other cytokines, such as IL-2 [28], IL-12 [29], IL-15 [30, 31], IL-17 [32], and IL-23 [33], play important roles in the induction of effector T cells and in the proliferation and IFN-*γ*-production of splenic T cells.

At 32 weeks after infection, compared to control mice, anti-IL-6R Ab-treated mice had similar TB CFU counts in the lungs but higher counts in liver and spleen (data not shown). This is consistent with the finding of Appelberg et al. that IL-6 inhibition by anti-IL-6 Ab did not affect the early stages of TB infection, but it did affect the later stages of it [15]. The increased growth of TB in anti-IL-6R Ab-treated mice at later stages of infection may be due to decreased effector T cell activity (Figure 5). As we and others [26, 27] have reported, IL-6 is an important cytokine in the induction of effector T cells, which play important roles in defense mechanisms against TB infection. In addition, the suppression of IFN-*γ* production in spleen cells observed with IL-6 blockade may also contribute to increased susceptibility to TB infection, since it has been reported that IL-6 [34] and IFN-*γ* inhibited the growth of *Mycobacterium bovis *in macrophages in vitro and that TB-infected IFN-*γ* KO mice and IFN-*γ* R mutant mice were unable to restrict the growth of TB and developed a fatal disseminated form of disease [35].

For comparison, we also investigated the effects of TNF-*α* blockade. Our findings confirmed that treatment of mice with anti-TNF-*α* antibody enhanced the growth of TB in the liver, spleen, and lungs, shortening the duration of survival (Figures 2 and 3), as previously reported [11]. A number of immature granulomata were observed in the lungs, spleen, and liver of anti-TNF-*α* Ab-treated mice (Figure 4(a)–4(c)). These results are consistent with previously published findings [10, 11, 35–39]. Since it has been reported that stimulation of fibroblast growth by TNF-*α* is required for the formation of granulomata that contain TB, sensitized effector T cells and activated macrophages that regulate the growth of TB, the aggravation of tuberculosis in anti-TNF-*α* Ab-treated mice is thought to be related to defective granuloma formation or apoptosis and necrosis of granuloma and lung structure [9–11, 35, 37–39].

We previously reported the efficacy of DNA vaccine combinations expressing mycobacterial heat shock protein 65 (Hsp65) and IL-12 in protecting against the emergence of IFN-*γ*-secreting T cells and activation of proliferative T cells and cytokine (IFN-*γ* and IL-2) production upon stimulation with Hsp65 and antigens from M. tuberculosis [19] and that vaccination with recombinant bacteria carrying expression plasmids for Ag85A, Ag85B, or MPB/MPT51 was capable of inducing specific cellular immune responses, such as proliferative responses of splenocytes and IFN-*γ* production by splenocytes [20]. These findings indicate that two functions, IFN-*γ* production and T cell proliferation, are significant in evaluating the effects of antimycobacterial drugs on immunity against mycobacteria. Anti-TNF-*α* Ab more potently inhibited the proliferation of splenic T cells and the production of IFN-*γ* in spleen cells than did anti-IL-6R Ab (Figure 5). These findings strongly suggest that TNF-a plays more important roles in mycobacteria immunity. One possible role of it is inhibition of the recruitment of immune cells into the spleen, since TNF-*α* plays an important role, via induction of chemokines, in recruiting immune cells, including effector T cells [40]. Another is that anti-TNF-*α* Ab treatment induces a systemic immunological defect, resulting from exhaustion of the body due to heavy TB infection. Taken together, these findings suggest that the increase in susceptibility to TB infection in anti-TNF-*α* Ab-treated mice can be attributed to malformation of granulomata and decreased effector T cell functions.

Since IL-6 KO and TNFR1 KO mice can be considered models of IL-6 and TNF-*α* blockade, we examined TB-challenged KO mice to supplement our investigation of Ab-treated mice. Although there were no significant differences between IL-6 KO and WT mice with respect to TB CFU counts in liver, lungs, or spleen 4 weeks after TB infection (Figure 6(b)), the shorter survival time of IL-6 KO mice (Figure 6(a)) shows that they have increased susceptibility to TB infection. It should be noted, however, that they survived longer than TNFR1 KO mice, and that they had higher TB-antigen-specific splenic T cell function than TNFR1 KO mice, as assessed by IFN-*γ* production (Figure 7). These findings of the experiment using KO mice also support the conclusion that TNF-*α* is a pivotal cytokine that plays a more important role than IL-6 in the protection of mice from TB infection.

It should be noted that in the case of TNFR1 KO mice, although levels of IFN-*γ* (Figure 7) and IL-2 (data not shown) in culture supernatant of PPD-stimulated spleen cells were markedly lower than for control WT mice, IL-6 level was only about 30% lower. This suggests that Th1 cell function may have been reduced, although IL-6-producing cell function was relatively well preserved in TNF-*α*-deficient mice infected with TB. In addition, even though IL-6 functions normally, TNF is required to protect hosts from TB infection.

Although anti-IL-6R Ab treatment did not decrease survival time (Figure 2), IL-6 KO mice died earlier than WT mice (Figure 6(a)). The following may explain the difference in survival results for anti-IL-6R Ab-treated and IL-6 KO mice. (1) IL-6 KO mice have fewer T lymphocytes and neutrophils than WT mice [41–43], and this can be expected to affect host defence against TB infection. (2) All WT DBA/1 mice died within 130 days after challenge with 5 × 10^5^ CFU of TB (Figure 6(a)), whereas BALB/c mice survived longer under the same conditions (Figure 2), suggesting that differences among strains exist in susceptibility to TB. It is possible that lack of IL-6 affected survival in the more susceptible strain of mice. (3) It is possible that the dose of anti-IL-6R Ab injected in this experiment was not sufficient to completely suppress IL-6 function. This seems unlikely, however, since SAA production was almost completely suppressed (Table 1) [24], and several published papers support the dose of anti-IL-6R Ab used in this experiment [44, 45]. (4) Differences among strains may be involved. In the antibody-treatment study, BALB/c mice were used, while in the KO mouse study DBA/1 mice were examined. Stronger susceptibility of DBA/1 mice to TB infection may have given rise to the discrepancy in findings.

Interestingly, anti-TNF-*α* Ab-treated mice exhibited increased levels of SAA protein. This finding contradicts previously reported results indicating that treatment of patients with TNF-*α* blockers decreased serum levels of acute-phase proteins such as C-reactive protein and SAA [1–3]. This discrepancy might be explained by increased growth of TB causing more severe inflammation and a resultant increase in acute-phase reaction.

Regarding whether the murine i.v. infection model we used fully reflects human TB, it should be noted that we have recently demonstrated that TB vaccination was as effective in this murine model as in a cynomolgus monkey intratracheal infection model [19, 46, 47]. Nevertheless, examination of the effects of Ab treatments in a model of reactivation of TB from the latent state may be even more clinically useful.

In summary, the results of these experiments all demonstrated that anti-IL-6R Ab treatment (and IL-6 KO status) had much less effect than anti-TNF-*α* Ab treatment (or TNFR1 KO status) on the development of TB infection in mice, suggesting that TNF-*α* is much more important than IL-6 in defence against TB infection, and that there may be less need to limit the clinical use of IL-6 blockers out of fear of reactivating TB.

##  Key Messages 

(1) TB-infected mice survived longer with less progression of infection with IL-6R blockade than with TNF-*α* blockade. 

(2) IL-6R blockade affected immune response to TB in mice less than TNF-*α* blockade.

##  Conflict of Interests 

T. Kishimoto is a professor at a research laboratory donated by Chugai Pharmaceutical. Y. Uchiyama, M. Mihara, and Y. Ohsugi are employees of Chugai Pharmaceutical Co. Ltd.

## Figures and Tables

**Table 1:** Serum amyloid A levels in BALB/c mice 4 weeks after challenge with TB.

Treatment			Serum amyloid A (*μ*g/mL)*
*M. tuberculosis*	Antibody	*n*	
(−)	(−)	5	15.1 ± 1.8
(+)	Control Ab 1	5	29.8 ± 1.3
(+)	Anti-IL-6R Ab	5	13.4 ± 1.3**^†^**
(+)	Control Ab 2	5	33.6 ± 6.9
(+)	Anti-TNF-*α* Ab	5	>189

*Values are means ± S.E.M.

^†^Significantly different (*P* < .01) from value for control Ab 1 (IL-6R)-treated group on Student's *t*-test.
